# Financing universal health coverage—effects of alternative tax structures on public health systems: cross-national modelling in 89 low-income and middle-income countries

**DOI:** 10.1016/S0140-6736(15)60574-8

**Published:** 2015-05-14

**Authors:** Aaron Reeves, Yannis Gourtsoyannis, Sanjay Basu, David McCoy, Martin McKee, David Stuckler

**Affiliations:** a Department of Sociology, University of Oxford, Oxford, UK; b London School of Hygiene & Tropical Medicine, London, UK; c Department of Medicine, Stanford University, Stanford, CA, USA; d Department of Primary Care and Public Health, Queen Mary University of London, London, UK

## Abstract

**Background:**

How to finance progress towards universal health coverage in low-income and middle-income countries is a subject of intense debate. We investigated how alternative tax systems affect the breadth, depth, and height of health system coverage.

**Methods:**

We used cross-national longitudinal fixed effects models to assess the relationships between total and different types of tax revenue, health system coverage, and associated child and maternal health outcomes in 89 low-income and middle-income countries from 1995–2011.

**Findings:**

Tax revenue was a major statistical determinant of progress towards universal health coverage. Each US$100 per capita per year of additional tax revenues corresponded to a yearly increase in government health spending of $9·86 (95% CI 3·92–15·8), adjusted for GDP per capita. This association was strong for taxes on capital gains, profits, and income ($16·7, 9·16 to 24·3), but not for consumption taxes on goods and services (−$4·37, −12·9 to 4·11). In countries with low tax revenues (<$1000 per capita per year), an additional $100 tax revenue per year substantially increased the proportion of births with a skilled attendant present by 6·74 percentage points (95% CI 0·87–12·6) and the extent of financial coverage by 11·4 percentage points (5·51–17·2). Consumption taxes, a more regressive form of taxation that might reduce the ability of the poor to afford essential goods, were associated with increased rates of post-neonatal mortality, infant mortality, and under-5 mortality rates. We did not detect these adverse associations with taxes on capital gains, profits, and income, which tend to be more progressive.

**Interpretation:**

Increasing domestic tax revenues is integral to achieving universal health coverage, particularly in countries with low tax bases. Pro-poor taxes on profits and capital gains seem to support expanding health coverage without the adverse associations with health outcomes observed for higher consumption taxes. Progressive tax policies within a pro-poor framework might accelerate progress toward achieving major international health goals.

**Funding:**

Commission of the European Communities (FP7–DEMETRIQ), the European Union's HRES grants, and the Wellcome Trust.

## Introduction

Universal health coverage (UHC) seems likely to feature in the post-2015 Millennium Development Goals.^1^, ^2^ In 2005, all 192 member states of the WHO committed themselves to achieving UHC, whereby “all people obtain the health services they need without suffering financial hardship when paying for them.”^3^ In 2012, Margaret Chan told the World Health Assembly that “Universal health coverage is the single most powerful concept that public health has to offer.”^4^ Subsequently, the UN General Assembly's resolution on UHC passed unanimously.^5^

Despite increasing recognition that UHC is an urgent social goal, only 20 low-income and middle-income countries (LMICs) currently have verifiable UHC.^6^ How best to achieve UHC remains unclear, especially in LMICs where opportunities for domestic financing might be constrained. The 2010 World Health Report set out a “Path to Universal Health Coverage”,^1^, ^2^ containing four financing strategies, such as increasing efficiency of taxation, reprioritising government budgets toward health, evaluating innovative financial mechanisms (eg, financial transaction taxes), and increasing development assistance for health. The 2013 World Health Report says that “the general solution for achieving wide coverage of financial risk protection [from financial ruin or impoverishment due to health-care costs] is through various forms of prepayment for services”.^2^ Recently, *The Lancet*'s Commission on Investing in Health called for a “grand convergence”^7^ in health with implementation of UHC. It recommended raising revenues through taxation (principally on tobacco and other unhealthy products) but failed to specify how sufficient revenue could be raised, particularly in highly deprived settings where a large fraction of health spending comes from donor assistance.^7^

Tax revenues are the main source of government funds available for financing and expanding health systems in most nations.^8^ In LMICs, tax revenues account for roughly 65% of total government revenues.^9^ However, few studies have rigorously evaluated how different tax regimes affect health systems.^10^ Although tax revenues can come from multiple sources, including corporate earnings, capital gains, profits, income, and consumption, most revenue growth in LMICs since 1990 has been from consumption taxes. Large informal economies create situations in which taxing goods and services might be a more feasible and stable source of government revenue than taxing income or wealth directly. Additionally, some economists, concerned that high taxes on profits or capital gains might deter foreign (or private) investment, have promoted indirect taxation, such as on consumption.^11^ Panel 1 describes three categories of tax on which the World Bank collects data, derived from the International Monetary Fund (IMF)'s Government Finances Statistics Manual.^12^, ^13^ Although the effect of different tax systems can vary substantially, in general, consumption taxes (taxes on goods and services) tend to be more regressive, placing a greater burden on low-income groups (panel 2 defines these terms).^14^, ^15^ Consumption taxes on staple food products and health-care services increase their overall price, so reducing utilisation just like user charges or copayments. For example, tax simulation models from South Africa find that although increased taxes on either general income, consumption, or both, could expand health coverage, consumption taxes would reduce access to nutrition and health-care for poorer households.^16^ However, the focus of much research and policy interest in health-care financing in LMICs has been on the scale of external aid and displacement of domestic spending, rather than on how domestic public finance is generated and how it can be increased.^17^, ^18^

Panel 1 **Forms of tax revenue reported by the World Bank**

Our analysis focuses on three main forms of tax as categorised and reported by the World Bank:

**Taxes on income, profits, and capital gains**

Taxes on income, profits, and capital gains are levied on the actual or presumptive net income of individuals, on the profits of corporations and enterprises, and on capital gains, whether realised or not, on land, securities, and other assets

**Taxes on goods and services**

Taxes on goods and services include general sales and turnover or value added taxes, selective excises on goods, selective taxes on services, taxes on the use of goods or property, taxes on extraction and production of minerals, and profits of fiscal monopolies

**Other taxes**

Other taxes include employer payroll or labour taxes (non-income), taxes on property, and taxes not allocable to other categories, such as penalties for late payment or non-payment of taxes

Panel 2 **Key tax-related definitions**

**Domestic tax revenue**

The sum of the charges or other levies imposed on taxpayers (both individuals and legal entities) by a state.

**Progressive taxation**

A progressive tax is one that takes a higher share of the income of the rich. An example is an income tax levied at 20% on the first £25 000, at 40% on all income between £25 000 and £50 000, and 60% at everything over £50 000.

**Regressive taxation**

A regressive tax is one that takes a higher share of the income of the poor. An example is a sales tax levied at 20% on basic amenities. Poor people typically pay a much higher share of their income on such amenities than rich people, so the total tax they pay will also be a much larger share of their income.

In this paper, for the first time to our knowledge, we test associations between alternative types of tax revenue and indicators designed to capture dimensions of the breadth, depth, and height of UHC.^1^, ^19^ These measures correspond, in turn, to the proportion of the population with access to health care, the scope of services covered, and their quality. We postulate that pro-poor or redistributive tax policies are likely to accelerate progress towards UHC.^20^

## Methods

### Data collection

We obtained data for tax revenues, health spending, gross domestic product (GDP), and development assistance for health from the World Bank's 2013 World Development Indicators,^13^ from 1995 to 2011. Tax revenues are defined as “the sum of all flows that are classified as taxes”, which specifically excludes “fines, penalties, and most social security contributions” such as compulsory social health insurance contributions. Health expenditure was disaggregated into government and private sources. Public health expenditure consists of social (or compulsory) health insurance funds including donations from international agencies. Private health expenditure includes direct household spending (out-of-pocket expenditure, OOP), private voluntary insurance, and other non-state forms of funding (eg, charities). All economic data were adjusted for inflation and purchasing power.

### Measuring dimensions of UHC

With no comparable single indicators of UHC, we draw on a series of measures that capture the breadth, depth, and height of coverage.^6^ We measured breadth using the International Labour Organization's indicator of financial coverage^21^ (the proportion of the population who would incur severe financial costs in accessing health care) for the latest available year. We measured access to care (depth of coverage) using the few cross-national data sources on health-care coverage, including the proportion of pregnant women receiving at least four antenatal visits and the proportion of births attended by a skilled health-care professional, again from the latest available year.^22^ Following previous studies^23^, ^24^ we assessed quality using outcomes data potentially amenable to health-care intervention including infant, neonatal, under-5 mortality per 1000 livebirths, and maternal mortality per 100 000 livebirths, taken from the Institute for Health Metrics and Evaluation (IHME), covering the years 1995–2011.^23^, ^24^

We compared three categories of taxation using World Bank taxation classification schema (panel 1): revenues from income, profits, and capital gains; revenues from taxes on the consumption of goods and services; and other forms of tax revenue, which include non-income labour taxes and fines. Although not all types of taxation are unequivocally progressive or regressive,^25^ the first tends to be more progressive, the second tends to be more regressive, and the third is ambiguous. Albeit an imperfect classification, reviews indicate that “sales [consumption] taxes are regressive wherever they are found”^19^, ^26^ and suggest that “the proportion of tax revenue raised through sales taxes can serve as an index of overall progressivity in situations where the detailed data…are not available”, as in many LMICs.^26^, ^27^, ^28^ The appendix shows descriptive statistics for all variables.

### Statistical models

To evaluate the association between tax revenue and health systems, we estimated a series of cross-national, multivariate ordinary least squares (OLS) models, correcting for country-specific differences (ie, country-specific slopes), as follows:

$$UHC_{it}=a+\beta_{1}\;Tax_{it}+B_{2}GDP_{it}+\mu_{i}+{ɛ}_{it}$$

Here, *i* is country and *t* is year. *UHC* is a vector of seven indicators of progress towards UHC. Four indicators are available on a longitudinal basis ([1] government health expenditure; [2] private health expenditure; [3] child mortality per 1000 livebirths including neonatal, post-neonatal, age 1–5 years, and under-5 mortality; and [4] maternal mortality per 100 000 livebirths). A further three were only available as a cross-section ([5] proportion of births with a skilled attendant; [6] proportion of women receiving at least four antenatal visits; and [7] proportion of the population who incur severe financial costs in accessing health care). We performed separate models for each of these seven dependent variables. *Tax* is the measure of tax revenue, similarly adjusted. Models included controls for GDP per capita, adjusted for inflation and purchasing-power parity. All models for which longitudinal data are available correct for linear country-specific time trends and country-specific fixed effects (μ); ɛ is the error term. Robust standard errors were clustered by country. In subsequent models we disaggregate total tax revenue into the three main World Bank categories mentioned above and include per capita development assistance for health to correct for the influence of aid programmes. All models were estimated using STATA version 12.

### Role of funding source

The funders of the study had no role in study design, data collection, data analysis, data interpretation, or writing of the report. The corresponding author had full access to all the data in the study and had final responsibility for the decision to submit for publication.

## Results

**Figure 1:**
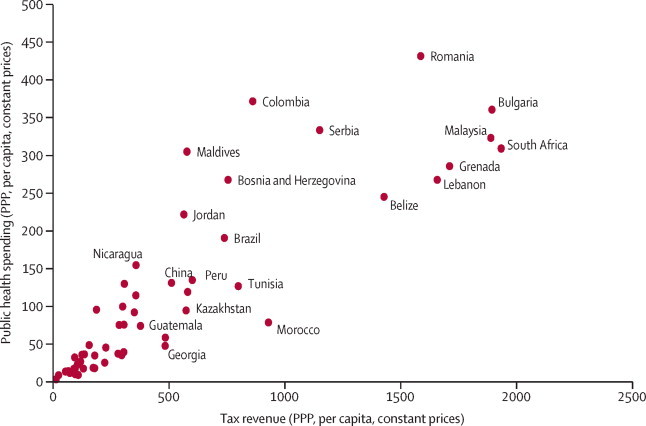
Tax revenues and health spending in low-income and middle-income countries, 2009 Source: World Bank Indicators. Excludes Botswana, Hungary, Seychelles, and St Vincent and Grenadines. Some countries are not labelled for sake of clarity. Tax revenue and public health spending is adjusted for inflation and purchasing power. PPP=purchasing power parity.

We noted a strong unadjusted association between tax revenues and government spending on health in 2009 (*r*=0·91, p<0·0001; figure 1). Table 1 shows results from the cross-national, fixed effects longitudinal models of the association of tax, GDP, and health spending for the years 1995 through 2011, adjusted for potential confounding factors. In LMICs, each $100 per-capita increase in tax revenue was associated with an additional public health spending per capita of $9·86. Each $100 increase in GDP per capita was associated with an increase of $1·86. Although not reported in table 1, adjusting for tax revenues substantially attenuated the GDP coefficient for public spending, consistent with taxation occupying a position on the pathway linking them (unadjusted β_GDP_=$3·98, 95% CI 2·38–5·58).

**Table 1:** Association of US$100 per capita increase in tax revenues and in GDP with health system expenditure in low-income and middle-income countries, 1995–2011

	Public health spending*		Private health spending*	
	Model 1	Model 2	Model 3	Model 4
$100 increase in tax revenue*	$15·8† (10·82 to 20·73; 2·49); p<0·0001	$9·86† (3·92 to 15·8; 2·99); p=0·0014	$4·95† (3·07 to 6·84; 0·95); p<0·0001	−$1·82 (−4·85 to 1·20; 1·52); p=0·23
$100 increase in GDP*	..	$1·86† (0·73 to 2·98; 0·57); p=0·0015	..	$2·12† (1·29 to 2·96; 0·42); p<0·0001
Number of countries	89	89	89	89
Country-years	813	813	813	813
*R*^2^	0·57	0·60	0·20	0·37

Source: World Bank Indicators. Models 1 and 2 have public health spending as the dependent variable. Models 3 and 4 have private health spending as the dependent variable. 95% CI and SEs are in parentheses and are adjusted for repeated observations. All models correct for country-specific differences and time trends. GDP=gross domestic product.

* Adjusted for purchasing power parity and inflation, per capita.

† p value <0·01.

We repeated these analyses for private health spending (table 1). Economic growth significantly associated with greater private health spending in LMICs; tax revenues, however, had no effect on private health spending after adjusting for economic growth rates.

In table 2 we evaluated how these associations differed by type of taxation. Using longitudinal fixed-effects models, we noted that each $100 rise in taxation is correlated with an $16·70 increase in government health expenditure from income, profits, and capital gains, whereas neither consumption taxes (−$4·37) nor other forms of taxation (−$3·40) were strongly associated with health spending. This pattern reflected the political economy of taxation; using data from the World Bank's Political Institutions database, we found that, after adjusting for GDP, governments ruled by left-leaning parties (categorised on the basis of the economic ideology of manifestos) raised, on average, $100·90 (95% CI 39·1 to 162·8) per capita greater tax revenues than those ruled by right-leaning parties (p=0·001), with a higher share of progressive sources, from income, profits, and capital gains (23·2% of tax revenues in left-leaning countries *vs* 19·5% of tax revenues in right-leaning countries). This was one reason why left-leaning parties tended to invest, on average, $23·2 (7·08 to 39·37) per person more in health than did right-leaning governments.

**Table 2:** Tax regime and government spending on health in low-income and middle-income countries, 1995–2011

	**Public health spending**		
	Model 1	Model 2	Model 3
$100 increase in tax revenue from income, profits, and capital gains (progressive)*	$22·1 (9·60 to 34·54; 6·27); p=0·0007	$22·9 (12·14 to 33·66; 5·41); p=0·0001	$16·7† (9·16 to 24·3; 3·80); p<0·0001
$100 increase in tax revenue from goods and services (regressive)*	..	−5·46% (−12·75 to 1·82; 3·67); p=0·14	$–4·37 (−12·9 to 4·11; 4·27); p=0·31
$100 increase in tax revenue from other taxes*	..	..	$–3·40 (−24·2 to 17·4; 10·4); p=0·75
Number of countries	88	88	86
Country-years	806	800	745

Source: World Bank Indicators and Organisation for Economic Co-operation and Development. Models 1 to 3 have public health spending as the dependent variable. Data are in US$ unless otherwise specified. 95% CIs and SEs are in parentheses and are adjusted for repeated observations. All models correct for country-specific differences and time trends.

* Adjusted for purchasing power parity and inflation, per capita.

† p value <0·01.

**Figure 2:**
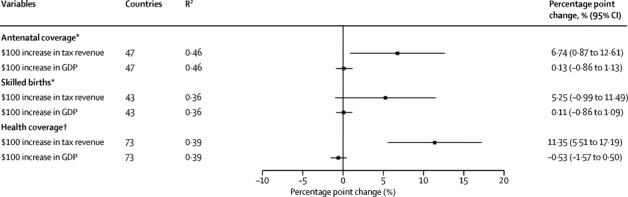
Tax revenues, GDP, and the breadth and depth of coverage in low-tax revenue countries Source: WHO, World Bank Indicators and International Labour Organization. All models estimated using ordinary least squares. Low tax revenue countries are those where revenues are less than $1000 per capita. Tax revenue and GDP are adjusted for purchasing power parity and inflation, per capita. GDP=gross domestic product. *Proportion of pregnancies. †Proportion of the population not exposed to severe financial costs. For full models see the appendix.

For the study of taxation and progress towards UHC, we first present unadjusted cross-sectional associations of tax revenues per capita with public health spending per capita, the proportion of populations with antenatal coverage, attended births, and post-neonatal mortality in the appendix. These show a clear convergence as tax revenues increase to the maximum 100% coverage. Since countries with high coverage and tax resources would be unlikely to gain additional benefit, unadjusted ordinary least squares would tend to underestimate the association between tax revenues and UHC. Thus we next evaluated the association of changes in tax revenues in low-revenue nations (ie, tax revenue <$1000 per capita per year) using cross-sectional models (figure 2). The appendix shows that each $100 increase in tax revenue was associated with a 6·74 percentage point (95% CI 0·87–12·6) increase in the proportion of women whose births were attended by a skilled worker and an 11·4 percentage point (5·51–17·2) increase in the proportion of the population with access to health insurance. After adjusting for GDP, we noted no association between tax revenues and maternal mortality (appendix)

The aforementioned statistical models show that tax revenues are strongly associated with greater investment in public health, access to services and better outcomes. However, some forms of taxation might have unintended consequences, adversely affecting low-income groups, especially when levied on goods and services, such as food and health care, necessary for survival. Thus, consumption taxes on health care are simply user charges that will reduce access and utilisation.^29^ Thus, to identify potential adverse consequences, we test the effectiveness of alternative tax regimes on health outcomes, holding constant the positive associations of tax revenues with greater health spending.

**Figure 3:**
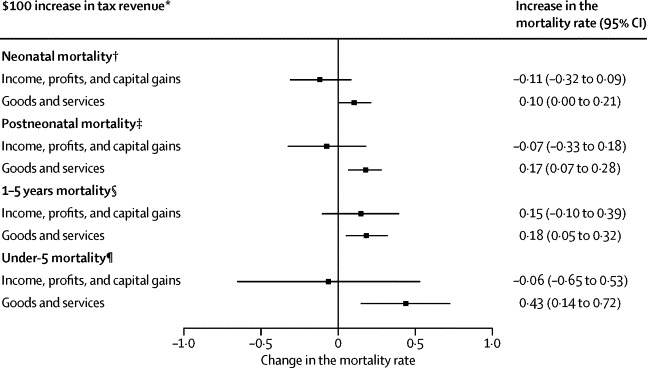
After correcting for health spending, the association of alternative tax regimes with child survival, 89 low-income and middle-income countries, 1995–2011 Source: World Bank Indicators and IHME. SEs are adjusted for repeated observations. All models correct for country-specific differences and time trends. The natural log of the dependent variable is used in these models. All models adjust for total public health spending and other tax revenue. *Adjusted for purchasing power parity and inflation, per capita. †Deaths per 1000 livebirths per year (before age of 1 month). ‡Deaths per 1000 livebirths per year (before the age of 1 year). §Deaths per 1000 livebirths per year (after the age of 1 year and under the age of 5 years); ¶Deaths per 1000 livebirths (under the age of 5 years). For full models see appendix.

After correcting for the association between infant mortality and government health spending, we evaluated the adjusted association of taxation with under-5 mortality. The forest plot (figure 3) shows the results of these four models. Each $100 increase per capita in revenues from consumption taxes, tending to reduce the ability of the poor to afford essential goods, was associated with a significantly higher post-neonatal mortality rate by 0·17 (95% CI 0·07–0·28), infant mortality rate (1–5 years) by 0·18 (0·05–0·32) and under-5 mortality rate by 0·43 (0·14–0·72). Taxes on income, profits, and capital gains were not associated with child survival outcomes (appendix).

Although consumption taxes are widespread, 13 governments had exemptions for health care and staple foods (appendix). These exemptions should mitigate the adverse effects described above. Using VAT data from Price Waterhouse Coopers, we found that, in the absence of tax exemptions for health care, consumption taxes, and infant mortality are even more strongly associated (0·50%, 0·18 to 0·83) whereas this association disappears where health care is exempt (0·23%, −2·59 to 3·05). A similar pattern was observed with staple foods.

Having examined the association between GDP and taxes, we examined one further source of revenue: aid inflows. Since ministries of finance tend to reduce health expenditures from domestic sources after receiving aid, particularly if receiving IMF loans,^17^ it might be possible that the inverse occurs, whereby greater taxes crowd out aid. We added to the models a covariate for overall development assistance per capita from the World Bank Development Indicators 2013. As shown in the appendix, there was no observed association of development assistance with health spending, whether public or private, and the associations of tax and GDP did not change for these outcomes.

We also did a series of robustness checks, finding consistent results (appendix).

## Discussion

Our findings suggest three main conclusions. First, in LMICs tax revenue is positively associated with progress towards UHC. Although there are examples of good health at low cost, UHC cannot easily be achieved without greater investment in health. Second, the commonly observed association of GDP with health spending seems largely mediated by greater tax revenues, increasing public, but not necessarily private health spending. Third, consumption taxes, which tend to be regressive, were adversely associated with child survival whereas there was no association with child survival and taxes on capital gains, profits, and incomes. This association was stronger in countries that taxed health care and staple foods.

As with any cross-national analyses, our study has important limitations, some reflecting (un)availability of data (panel 3). There is potential for ecological fallacies; however, all observed associations are plausible, with well documented mechanisms.^30^ We did not have longitudinal data for some indicators of UHC, particularly antenatal care access and financial coverage, emphasising the need to improve health-system surveillance in resource-deprived settings and to develop “internationally comparable indicators of quality.”^2^ Further, the International Labour Organization (ILO) social health protection indicator probably overestimates coverage by private insurance whereas the ILO financial coverage indicator measures total spending rather than a core set of efficient services. Although we used the best available mortality data reporting inaccuracies could have occurred, which could yield conservative estimates of the link between taxes and health systems. Without equity-based metrics of UHC, the current country-level UHC measures might mask within-country inequalities, especially in large countries such as China or India.^31^

Panel 3 **Research in context**

**Systematic review**

We initially searched PubMed up to Jan 1, 2015, using search terms “tax” AND “universal health coverage”. This yielded a total of 12 studies, all which were policy analyses, rather than research studies. Of these, seven papers argued that tax-based financing was necessary for progress toward UHC in Latin America, Canada, Kenya, Costa Rica, and low-middle-income countries. The other five did not have tax as a central component but commented on the health consequences of policy making. One empirical study used tax registry data to link sociodemographic data with patient outcomes. None of these studies quantified the relation between the tax and progress towards UHC. We extended our search in JSTOR and found a recent review (2014) of the political, economic, and social effects of taxation which concluded that “there is as yet little research on how taxation affects factors such as infant mortality”. There were no studies identified which comparatively or systematically evaluated the impact of taxation on progress toward universal health coverage or to reducing mortality in low-income and middle-income countries.

**Interpretation**

There is intense debate on how to finance progress towards UHC in low-income and middle-income countries. Our study advances previous work by investigating how alternative tax systems affect the breadth, depth, and height of health system coverage. Using cross-national longitudinal fixed effects models, we noted that tax revenue was a major statistical determinant of progress towards UHC. Each US$100 per capita per year of additional tax revenues corresponded to a $9·86 yearly increase in government health spending, adjusted for GDP per capita. In countries with low tax revenues, an additional $100 tax revenue per year substantially increased the proportion of births attended by a skilled attendant by 6·74 percentage points and the extent of financial coverage by 11·4 percentage points. Consumption taxes, a more regressive form of taxation that may reduce the ability of the poor to afford essential goods, were associated with significant higher post-neonatal mortality, infant mortality, and under-5 mortality. These adverse associations were not seen with taxes on capital gains, profits, and income, which tend to be more progressive. Increasing domestic tax revenues is integral to achieving UHC, particularly in countries with low tax bases. Pro-poor taxes on profits and capital gains appear to support expanding health coverage without the adverse associations with health outcomes observed for higher consumption taxes.

Future research is also needed to assess how the political orientation of the government might affect both progressivity of taxation and allocation to health. Our results suggest that taxes on income, profits, and capital gains are positively associated with government health spending whereas consumption taxes are not. This finding portrays how left-leaning governments invest more than right-leaning governments in social protection programmes, including health, through general taxation, and draw taxes on income, profits, and capital gains that pose relatively less harm to deprived groups.^32^

The World Bank tax data have limitations, combining multiple sources into single measures. Future surveillance efforts must disaggregate these sources to enable comparison of their effectiveness. Our analysis used the average level of tax revenue per person and was unable to account for differing degrees of tax avoidance, more likely to occur among high-income groups and large multinational corporations.^33^ More work is also needed to understand how tax revenues can be increased in economies dominated by informal production and in settings in which costs of registering taxable activity are lower.^11^

Notwithstanding these limitations, our study has important implications for global health policy. First, development of a stable, tax-revenue base might help donor-dependent countries transition to independence from external aid financing. The UK government, for example, worked with two developing countries (Ethiopia and Tanzania) to reduce tax evasion, increasing tax revenues by 40% between 2010 and 2013.^34^ Such interventions could strengthen health systems. Before the Ebola outbreak in Sierra Leone, only one in five leading mining companies had paid any corporate income tax.^35^

Well chosen consumption taxes can serve population health, like those on cigarettes and alcohol, and others can be more progressive, such as taxing luxury goods. Second, although increasing GDP will, if tax rates remain stable, increase health spending, over the past decade we have not seen this pattern. As global GDP rose over the past decade, global tax revenues, as a proportion of global GDP, fell from 15·7% in 2001 to 13·6% in 2010, corresponding to a loss of US$1·4 trillion in revenue, enough to finance UHC at current estimates.^12^

Low-income countries have lower tax-to-GDP ratios than do high-income countries.^11^ Taking the Indian government as an example, in 2011 it spent $28 per person on health. If India increased tax revenue from 10·4% of GDP to 14·4%, the proportion seen in high-income countries, it would generate additional revenue of $44·3 per person—ample to finance Chatham House's UHC goals and attain *The Lancet*'s “grand convergence”^7^ in child and maternal mortality.^7^, ^28^ While raising already high taxes further might yield less revenue, the low tax rates in LMICs suggest a wide scope to increase them.^36^

In view of strong evidence that investing in health improves economic growth, progressive tax policies should enhance economic performance and reduce health inequalities between countries, while reducing poverty and promoting UHC.
