# Supplementary material for: Financing universal health coverage—effects of alternative tax structures on public health systems: cross-national modelling in 89 low-income and middle-income countries
Source: Lancet. 2015 May 14;386(9990):274–80. doi: 10.1016/S0140-6736(15)60574-8 (PMC4513966; doi:10.1016/S0140-6736(15)60574-8)
Supplement: Supplementary appendix [file mmc1.pdf]

THE LANCET

Supplementary appendix

This appendix formed part of the original submission and has been peer reviewed. We post it as supplied by the authors.

Supplement to: Reeves A, Gourtsoyannis Y, Basu S, McCoy D, McKee M, Stuckler D. Financing universal health coverage—effects of alternative tax structures on public health systems: cross-national modelling in 89 low-income and middle-income countries. *Lancet* 2015; published online May 15. http://dx.doi.org/10.1016/S0140-6736(15)60574-8.

Web Appendix

Web Appendix 1: Summary statistics

Web Appendix 2a: Antenatal coverage and tax revenue among 48 Low- and Middle-Income Countries

Web Appendix 2b: Proportion of births attended by skilled health personnel and tax revenue among 59 Low- and Middle-Income Countries

Web Appendix 2c: Post-neonatal mortality and tax revenue among 58 Low- and Middle-Income Countries, 2009

Web Appendix 3: Access to healthcare among low tax revenue countries

Web Appendix 4: Health coverage among low tax revenue countries

Web Appendix 5: Tax revenue, GDP, and maternal mortality among Low- and Middle-Income Countries, 1995-2011

Web Appendix 6: Tax regimes and mortality across Low- and Middle-Income Countries, 1995-2011

Web Appendix 7: VAT rate and exemptions among Low- and Middle-Income countries, latest available data

Web Appendix 8: Change in public health spending with change in Tax revenues, GDP, and Official Development Assistance (ODA), in Low- and Middle-Income Countries 1995-2011

Web Appendix 9: Robustness and Sensitivity Checks

Web Appendix 10: Change in public health spending with change in Tax revenues and GDP excluding outliers, in Low- and Middle-Income Countries 1995-2011

Web Appendix 11: Tax regimes and mortality across Low- and Middle-Income Countries excluding outliers, 1995-2011

Web Appendix 12: Tax revenue, GDP, and maternal mortality among Low- and Middle-Income Countries excluding outliers, 1995-2011

Web Appendix 13: Sims causality test, differenced specification

Web Appendix 14: Tax revenue, GDP, and out-of-pocket expenditure among Low- and Middle-Income Countries, 1995-2011

Web Appendix 15: Tax revenues, urbanization and UHC among Low- and Middle-Income Countries, latest available year

Web Appendix 16: After correcting for public health spending, private spending and aid, the association of alternative tax regimes with infant mortality, 86 low- and middle-Income countries, 1995-2011

Web Appendix 17: After correcting for the Great Recession, the association of alternative tax regimes with infant mortality, 86 low- and middle-Income countries, 1995-2011

Web Appendix 1: Summary statistics

Variable	Mean (SD)	Min	Max	Country-years	Countries	Coding	Source
GDP[1]	4031·65 (4174·81)	39·32	24567·02	803	89	PPP, constant, per capita	World Bank Indicators
Public Health spending[1]	135·24 (184·71)	0·87	1190·06	803	89	PPP, constant, per capita	World Bank Indicators
Private Health spending[1]	98·84 (99·22)	0·54	524·84	813	89	PPP, constant, per capita	World Bank Indicators
Antenatal coverage[2]	68·04 (23·52)	14·60	96·9	47	47	Proportion of pregnancies	WHO
Skilled Birth[3]	76·73 (25·72)	10·00	100·00	59	59	Proportion of births	WHO
Official Development Assistance[1]	47·77 (76·47)	-15·77	927·17	793	89	PPP, constant, per capita	World Bank Indicators
Tax revenue[1]	717·98 (980·28)	0·65	6676·87	813	89	PPP, constant, per capita	World Bank Indicators
Good & Service (Tax)[1]	310·31 (440·24)	1·03	2928·42	806	89	PPP, constant, per capita	World Bank Indicators
Other forms of Tax[1]	29·92 (48·93)	-101·91	317·43	756	87	PPP, constant, per capita	World Bank Indicators
Income, Profits and Capital Gains (Tax)[1]	910·69 (1137·52	1·03	6638·057	806	88	PPP, constant, per capita	World Bank Indicators
Neonatal mortality	20·67 (12·25)	2·74	58·13	759	89	Per 1,000 live births	IHME
Post-neonatal mortality	17·40 (13·59)	0·98	61·27	759	89	Per 1,000 live births	IHME
Infant mortality (1-5)	15·87 (21·66)	0·32	106·33	759	89	Per 1,000 live births	IHME
Under five mortality	52·41 (42·26)	4·04	199·64	759	89	Per 1,000 live births	IHME
Maternal mortality	226·61	9	1772·8	734	80	Per 100,000 births	IHME

	(272·36)						
Healthcare coverage	54·51 (37·94)	0·1	100	88	88	Proportion of the population	ILO

Notes:
1 – Per capita, adjusted for inflation and purchasing-power.
2 – Proportion of pregnancies where the mother receives four or more antenatal visits.
3 – Proportion of births attended by a skilled health professional

Web Appendix 2a: Antenatal coverage and tax revenue among 48 Low- and Middle-Income Countries

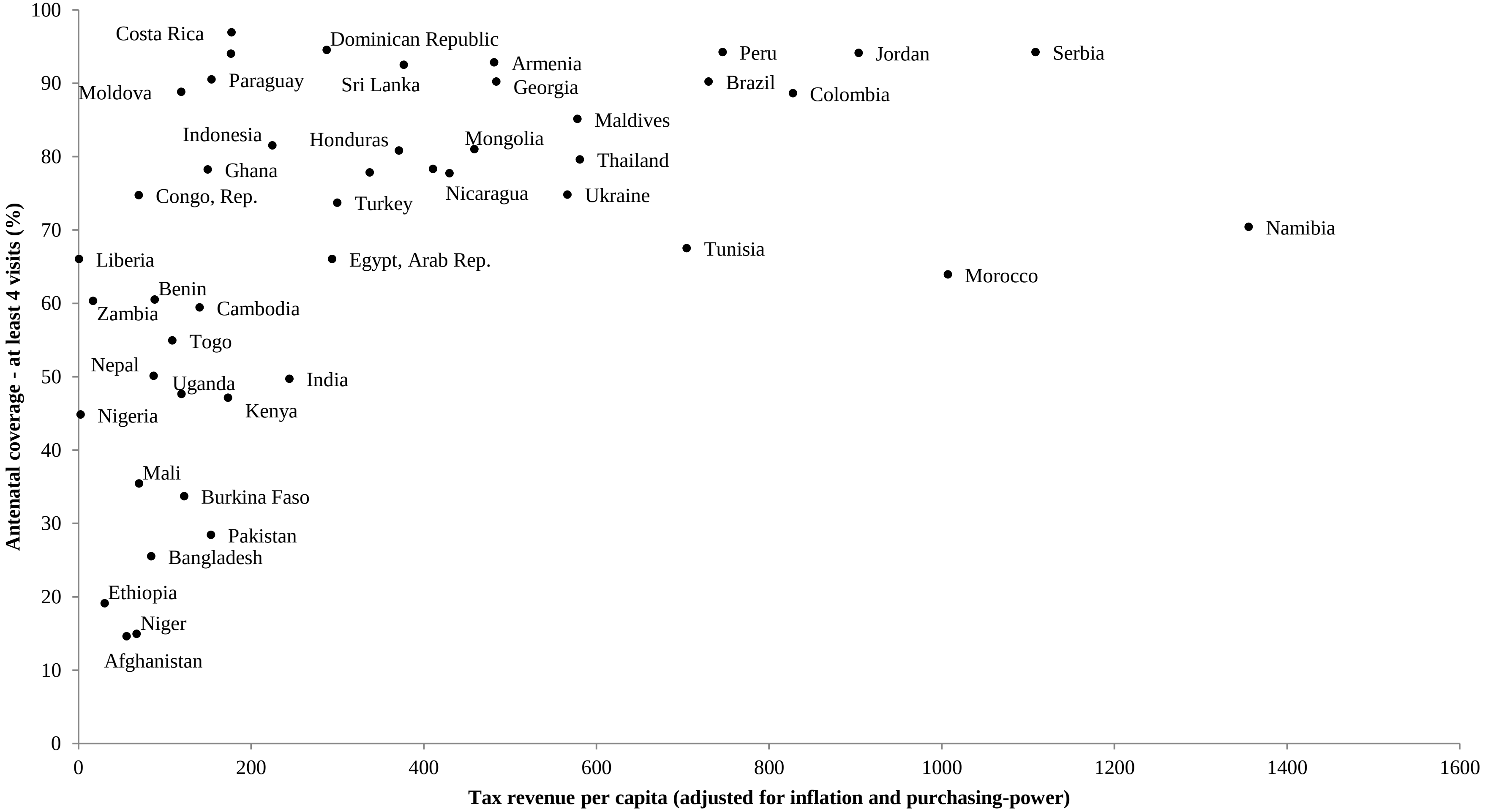

Notes: Source: World Bank Indicators and World Health Observatory. Some countries are not labelled for sake of clarity. Country observations are taken from different years (i.e., Brazil is 2010 while Kenya is 2009). Tax revenue is adjusted for inflation and purchasing-power. Some countries have very low (but non-zero) tax revenues and quite high levels of antenatal coverage. These countries also receive high levels of external aid.

Web Appendix 2b: Proportion of births attended by skilled health personnel and tax revenue among 59 Low- and Middle-Income Countries

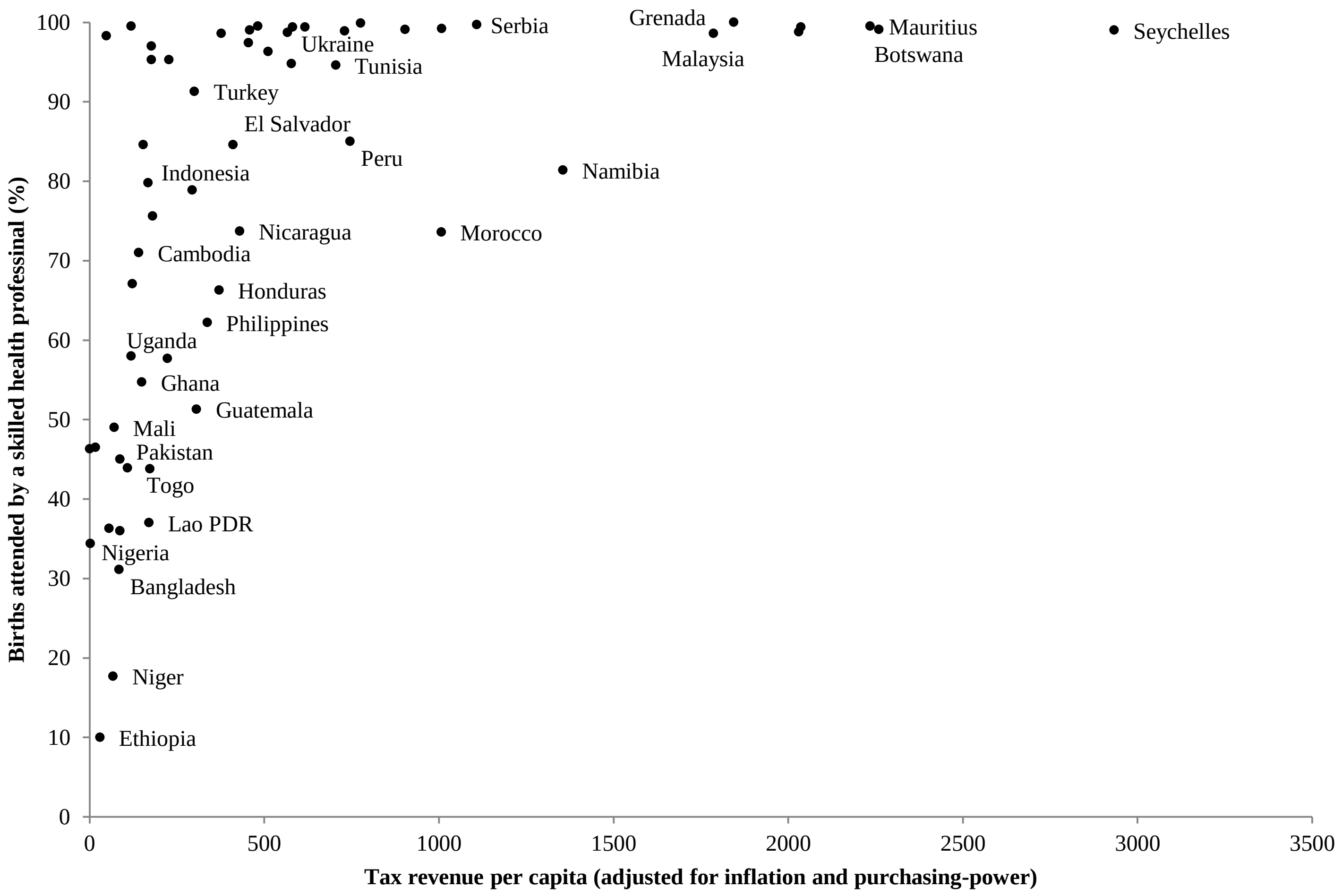

Notes: Source: World Bank Indicators and World Health Observatory. Excludes Hungary. Some countries are not labelled for sake of clarity. Country observations are taken from different years. Tax revenue is adjusted for inflation and purchasing-power..Some countries have very (but non-zero) low tax revenues and quite high levels of antenatal coverage. These countries also receive high levels of external aid.

Web Appendix 2c: Post-neonatal mortality and tax revenue among 58 Low- and Middle-Income Countries, 2009

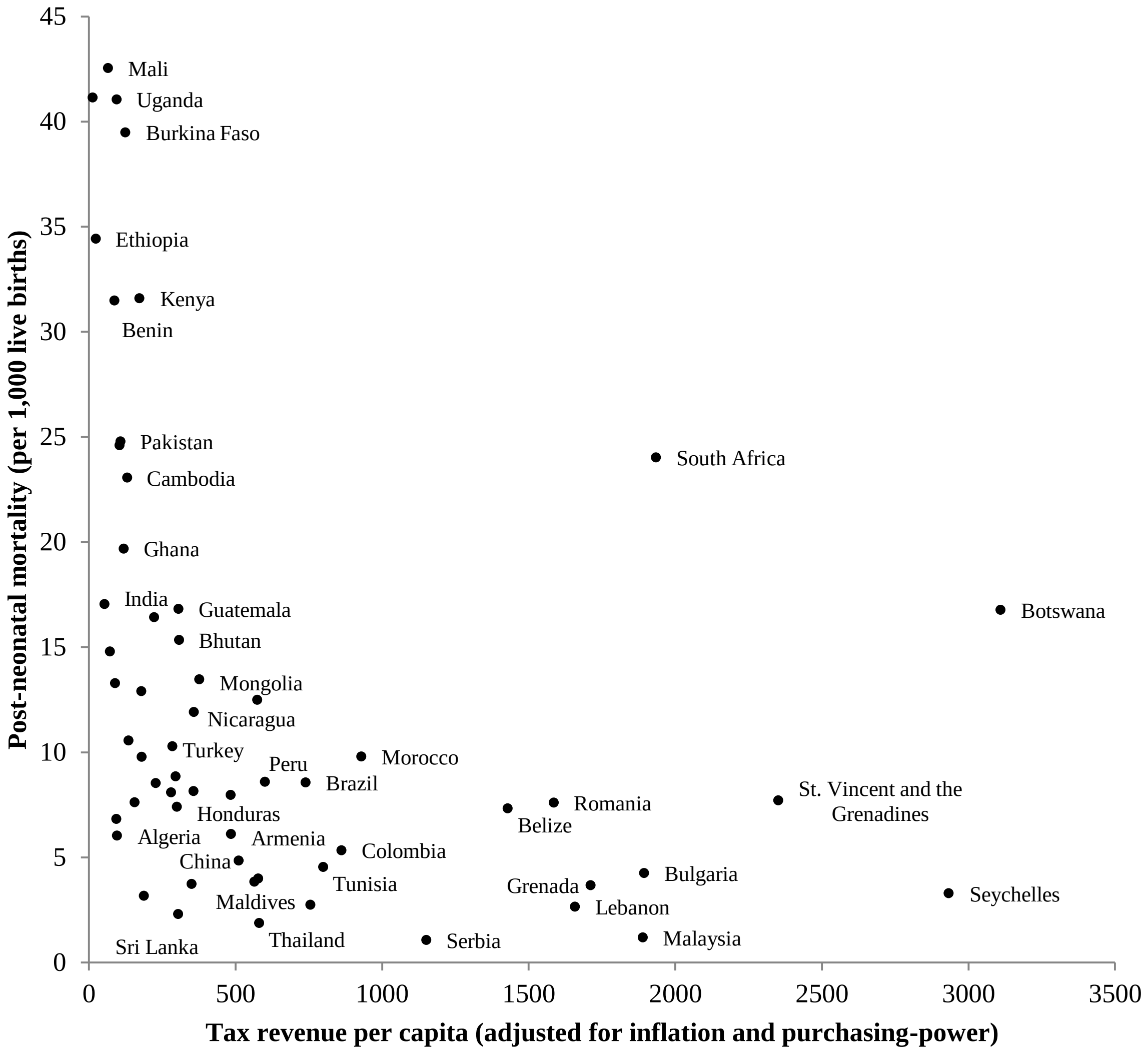

Notes: Source: World Bank Indicators and IHME. Some countries are not labelled for sake of clarity. Tax revenue is adjusted for inflation and purchasing-power.

Web Appendix 3: Access to healthcare among low tax revenue countries

**A. Antenatal coverage**	Antenatal coverage[1]	
	(1)	(2)
\$100 increase in Tax revenue[2]	5·91** (1·24)	5·25 (3·09)
\$100 increase in GDP[2]		0·11 (0·48)
Number of countries	43	43
R^2	0·36	0·36
**B. Skilled birth**	Skilled birth[1]	
	(1)	(2)
\$100 increase in Tax revenue[2]	7·46** (1·21)	6·74* (2·91)
\$100 increase in GDP[2]		0·13 (0·49)
Number of countries	47	47
R^2	0·46	0·46

Notes: Source: World Bank Indicators. Standard errors are in parentheses. All models estimated using OLS. * p-value < 0·05, ** p-value < 0·01. Low tax revenue countries are those where revenues are less than \$1000 per capita. 1 – Proportion of pregnancies 2 – Adjusted for purchasing power parity and inflation, per capita.

Web Appendix 4: Health coverage among low tax revenue countries

**Variables**	Health coverage (% of population)[1]	
	(1)	(2)
$100 increase in Tax revenue[2]	8·65** (1·28)	11·3** (2·93)
$100 increase in GDP[2]		-0·53 (0·52)
Number of countries	73	73
R^2	0·39	0·40

Notes: Source: World Bank Indicators. Standard errors are in parentheses. All models estimated using OLS. * p-value < 0·05, ** p-value < 0·01. Low tax revenue countries are those where revenues are less than $1000 per capita. 1 – Proportion of the population

Web Appendix 5: Tax revenue, GDP, and maternal mortality among Low- and Middle-Income Countries, 1995-2011

	Maternal mortality[1]	
	(1)	(2)
\$100 increase in Tax revenue[2]	-2·95*	-6·21
	(1·12)	(4·47)
\$100 increase in GDP[2]		1·08
		(1·34)
Country-years	734	734
Countries	80	80

Notes: Source: World Bank Indicators and IHME. Standard errors are in parentheses and are adjusted for repeated observations. All models correct for country-specific differences and time trends.
* p-value < 0·05, ** p-value < 0·01. 1 - Maternal mortality is number of maternal deaths per 100,000 births. 2 – Adjusted for purchasing power parity and inflation, per capita.

Web Appendix 6: Tax regimes and mortality across Low- and Middle-Income Countries, 1995-2011

**A. Neonatal Mortality**	Neonatal mortality[1]		
	(1)	(2)	(3)
$100 increase in tax revenue from income, profits, and capital gains (progressive)[5]	-0·05<br>(0·09)	-0·08<br>(1·00)	-0·12<br>(0·10)
$100 increase in tax revenue from goods and services (regressive)[5]		0·09<br>(0·05)	0·01<br>(0·05)
$100 increase in tax revenue from other taxes[5]			0·72<br>(0·50)
Number of countries	88	88	86
Country-years	752	746	697
**B. Post-neonatal Mortality**	Post-neonatal mortality[2]		
	(1)	(2)	(3)
$100 increase in tax revenue from income, profits, and capital gains (progressive)[5]	0·02<br>(0·12)	-0·04<br>(0·13)	-0·07<br>(0·13)
$100 increase in tax revenue from goods and services (regressive)[5]		0·16*<br>(0·06)	0·17**<br>(0·06)
$100 increase in tax revenue from other taxes[5]			0·73<br>(0·62)
Number of countries	88	88	86
Country-years	752	746	697
**C. Infant Mortality (1-5 years)**	Infant (1-5) mortality[3]		
	(1)	(2)	(3)
$100 increase in tax revenue from income, profits, and capital gains (progressive)[5]	0·28*<br>(0·14)	0·22<br>(0·13)	0·15<br>(0·13)
$100 increase in tax revenue from goods and services (regressive)[5]		0·18*<br>(0·08)	0·18**<br>(0·07)
$100 increase in tax revenue from other taxes[5]			0·70<br>(0·50)
Number of countries	88	88	86
Country-years	752	746	697
**D. Under five Mortality**	Under 5 mortality[4]		
	(1)	(2)	(3)
$100 increase in tax revenue from income, profits, and capital gains (progressive)[5]	0·22<br>(0·27)	0·07<br>(0·30)	-0·06<br>(0·30)
$100 increase in tax revenue from goods and services (regressive)[5]		0·41*<br>(0·16)	0·43**<br>(0·15)
$100 increase in tax revenue from other taxes[5]			2·06<br>(1·48)
Number of countries	88	88	86
Country-years	752	746	697

Notes: Source: World Bank Indicators and IHME. Standard errors are in parentheses and are adjusted for repeated observations. All models correct for country-specific differences and time trends. All models adjust for total public health spending. 1 – Deaths per 1,000 live births (before age of 1 month); 2 – Deaths per 1,000 per year (before the age of 1); 3 – Deaths per 1,000 per year (after the age of 1 and under the age of 5); 4 – Deaths

per 1,000 live births (under the age of 5); 5 – Adjusted for purchasing power parity and inflation, per capita. * p-value < 0·05, ** p-value < 0·01

Web Appendix 7: VAT rate and exemptions among Low- and Middle-Income countries, latest available data

Country	VAT rate	Health exemption	Staple goods exemption	Notes
Afghanistan				
Albania	20%	10%		
Algeria	17%			7% for basic items
Argentina	21%	X	X	Other items vary (27% or 10.5%)
Armenia	20%			
Azerbaijan	18%			
Bangladesh				
Belize				
Benin				
Bhutan				
Bolivia	13%			
Bosnia and Herzegovina	17%			
Botswana	12%			Some exemptions but no clear details
Brazil	10-15%			An additional state tax (7% to 25%)
Bulgaria	20%			
Burkina Faso				
Burundi				
Cameroon	19.25%			
Cape Verde	15%	X	X	
China	17%			
Colombia	16%	5%	X	
Congo, Rep.	18%			
Costa Rica	13%		X	
Dominican Republic	8% to 18%	X	X	
Egypt, Arab Rep.				10% = goods; 5%-10% = services
El Salvador	13%	X		
Ethiopia				
Fiji	15%		X	
Georgia	18%	X		
Ghana	15%			
Grenada				
Guatemala	12%			
Guinea				
Honduras	15%			
Hungary	27%		18%	5% for some pharmaceuticals
India				5% to 15% varies by State
Indonesia	10%			
Iran, Islamic				

Rep.				
Jordan	16%	X	X	
Kazakhstan	12%			
Kenya	16%			
Kyrgyz Republic	12%			
Lao PDR	10%	X		
Lebanon	10%			
Lesotho				
Liberia				
Macedonia, FYR	18%		5%	5% for pharmaceuticals
Madagascar	20%			
Malaysia	6%			
Maldives				
Mali				
Mauritius	15%	X	X	
Mexico	16%		X	Pharmecueticals are exempted
Moldova	20%		8%	8% for pharmaceuticals
Mongolia	10%			
Morocco	20%		X	
Namibia	15%			
Nepal				
Nicaragua	15%		X	Exemptions for medical products
Niger				
Nigeria	5%	X	X	
Pakistan	17%		X	
Panama	7%	X		
Papua New Guinea	10%	X		
Paraguay	10%		5%	5% for pharmaceuticals
Peru	18%			
Philippines	12%			
Romania	24%	9%		
Senegal	18%			
Serbia	20%			10% for medicines
Seychelles				
South Africa	14%			
Sri Lanka	12%			
St. Vincent and the Grenadines				
Sudan				
Swaziland	14%			
Syrian Arab Rep				

Tajikistan	18%			
Thailand	7%	X	X	
Togo				
Tunisia	18%			
Turkey	18%		8%	
Uganda	18%	X		Some foodstuffs
Ukraine	20%			
Vanuatu				
Venezuela, RB		X	X	
Yemen, Rep.				
Zambia	16%			

Notes: Source: Price Waterhouse Cooper country profiles. No data is available for Italicized countries. Where exemption cells contain an X the country implements a full exemption whereas if it is only a lower rate we report this lower rate where it is available.

Web Appendix 8: Change in public health spending with change in Tax revenues, GDP, and Official Development Assistance (ODA), in Low- and Middle-Income Countries 1995-2011

**All countries**	Public Health spending[1]			Private Health Spending[1]		
	(1)	(2)	(3)	(4)	(5)	(6)
$100 increase in Tax revenue[1]	15·8** (2·49)	9·86** (2·99)	11·4** (2·58)	9·37** (1·68)	1·00 (2·28)	0·11 (2·41)
$100 increase in GDP[1]		1·86** (0·57)	1·55** (0·51)		2·05** (0·43)	2·11** (0·46)
$100 increase in ODA[1]			2·61 (4·34)			-1·35 (4·28)
Number of countries	89	89	89	89	89	86
Country-years	813	813	793	750	750	740

Notes: Source: World Bank Indicators. Standard errors are in parentheses and are adjusted for repeated observations. All models correct for country-specific differences and time trends.

* p-value < 0·05, ** p-value < 0·01

1 – Adjusted for purchasing-power parity and inflation, per capita.

Web Appendix Text 9. Robustness and Sensitivity Tests

To address the impact of potential outliers we calculated the standardized residuals and then re-estimated the models excluding those observations with residuals >|2|. We observe that the association between tax and public health spending does not qualitatively change (Web Appendix 10). Further we also find that detrimental effect of consumption taxes on infant mortality is unchanged after excluding these outliers (see Web Appendix11) and that there is still no direct effect of tax revenue on maternal mortality (Web Appendix 12). To ensure the associations observed between tax, government health spending and GDP are not spurious, we use a Sims causality test to examine whether the prediction of health spending from past and present observations of tax revenue would be improved if future values of tax revenue are included in the model.[44] Here the joint-F test of present and past periods of tax and GDP are significant at $p<0{\cdot}0001$, whereas the future values are not ($p=0{\cdot}49$ and $p=0{\cdot}23$ respectively) (Web appendix 13). Because our measure of private spending combines both out-of-pocket (OOP) and other forms of non-government expenditure, we estimate the impact of GDP and tax revenue on out-of-pocket expenditure only, finding that our results do not qualitatively change (Web Appendix 14). Because access to maternal care and UHC may be greater in densely populated areas, we adjust our models for the degree of urbanization and find that, although urbanization is associated with greater coverage, the association with tax does not qualitatively change (Web Appendix 15). Infant mortality will be influenced by private health spending and aid in addition to public health spending and so we test whether the negative effects of consumption taxes on infant mortality persist after adjusting for these other forms of health spending; our results were consistent in this model specification (Web Appendix 16). Finally, since the 2008 global financial crisis might have created a break from past trends, we replicated models using a binary variable to capture a potential deviation from long-term trends for the years 2008-2011. Again, none of our basic findings was changed as shown in Web Appendix 17.

Web Appendix 10: Change in public health spending with change in Tax revenues and GDP excluding outliers, in Low- and Middle-Income Countries 1995-2011

	Public Health spending[1] (1)	Private Health Spending[1] (2)
$100 increase in Tax revenue[1]	8·06** (2·12)	-0·78 (1·65)
$100 increase in GDP[1]	1·73** (0·43)	1·91** (0·39)
Country-years	790	792
Countries	89	89

Notes: Source: World Bank Indicators. Standard errors are in parentheses and are adjusted for repeated observations. All models correct for country-specific differences and time trends. Outliers are those observations with standardised residuals >|2|.
* p-value < 0·05, ** p-value < 0·01
1 – Adjusted for purchasing-power parity and inflation, per capita.

Web Appendix 11: Tax regimes and mortality across Low- and Middle-Income Countries excluding outliers, 1995-2011

	Neonatal mortality[1] (1)	Post-neonatal mortality[2] (2)	Infant (1-5) mortality[3] (3)	Under 5 mortality[4] (4)
$100 increase in tax revenue from income, profits, and capital gains (progressive)[5]	-0·10 (0·082)	-0·17 (0·14)	0·084 (0·081)	-0·15 (0·26)
$100 increase in tax revenue from goods and services (regressive)[5]	0·097* (0·039)	0·16** (0·055)	0·14** (0·046)	0·39** (0·13)
$100 increase in tax revenue from other taxes[5]	0·66 (0·45)	0·80 (0·47)	0·60 (0·39)	1·97 (1·29)
Country-years	675	671	676	677
Countries	82	82	85	85

Notes: Source: World Bank Indicators and IHME. Standard errors are in parentheses and are adjusted for repeated observations. All models correct for country-specific differences and time trends. All models adjust for total public health spending. Outliers are those observations with standardised residuals >|2|. 1 – Deaths per 1,000 live births (before age of 1 month); 2 – Deaths per 1,000 per year (before the age of 1); 3 – Deaths per 1,000 per year (after the age of 1 and under the age of 5); 4 – Deaths per 1,000 live births (under the age of 5); 5 – Adjusted for purchasing power parity and inflation, per capita. * p-value < 0·05, ** p-value < 0·01

Web Appendix 12: Tax revenue, GDP, and maternal mortality among Low- and Middle-Income Countries excluding outliers, 1995-2011

	Maternal mortality[1] (1)
$100 increase in Tax revenue[2]	1·15<br>(2·53)
$100 increase in GDP[2]	-0·77<br>(0·52)
Country-years	724
Countries	78

Notes: Source: World Bank Indicators and IHME. Standard errors are in parentheses and are adjusted for repeated observations. All models correct for country-specific differences and time trends. Outliers are those observations with standardised residuals >|2|.
* p-value < 0·05, ** p-value < 0·01. 1 - Maternal mortality is number of maternal deaths per 100,000 births. 2 – Adjusted for purchasing power parity and inflation, per capita.

Web Appendix 13: Sims causality test, differenced specification

The Sims causality test asserts that "the prediction of y from current and past x's would not be improved if future values of x are included" (Maddala, Ch. 9 pg. 394). In other words, the future should not cause the past. According to Sims (1972), if you regress Y on past, present, and future values of X, then "if causality runs from X to Y only, future values of X in the regression should have coefficients insignificantly different from zero as a group" (Sims 1972, p. 545).

In the manuscript we followed standard practice by conducting a joint F-test for whether the future values were significant and added explanatory value over the present and current values (set of β-coefficients) to test this hypothesis.

We find that both future tax revenues and future GDP have no association with Health spending while past and present tax revenues and GDP do have a significant association. These results suggest that 'causality runs from X to Y' and not the other way around.

Lag and lead covariates	ΔHealth spending $_{t}$
ΔTax revenue $_{t, t-1}$ (Present and Past)	$F(2, 80) = 12{\cdot}48$**
ΔTax revenue $_{t+1}$ (Future)	$F(1, 80) = 0{\cdot}49$
Lag and lead covariates	ΔHealth spending $_{t}$
ΔGDP $_{t, t-1}$ (Present and Past)	$F(2, 88) = 20{\cdot}98$**
ΔGDP $_{t+1}$ (Future)	$F(1, 88) = 1{\cdot}44$

Notes: Source: World Bank Indicators. Standard errors are in parentheses and are adjusted for repeated observations. All models correct for country-specific differences and time trends. Joint-F tests are reported in the table. Results remain qualitatively unchanged if estimated with two lags and two leads. * p-value < 0·05, ** p-value < 0·01.

Web Appendix 14: Tax revenue, GDP, and out-of-pocket expenditure among Low- and Middle-Income Countries, 1995-2011

	Out-of-pocket expenditure on health[1] (1)
$100 increase in Tax revenue[1]	-$0·64<br>(1·27)
$100 increase in GDP[1]	$1·79**<br>(0·35)
Country-years	813
Countries	89

Notes: Source: World Bank Indicators. Standard errors are in parentheses and are adjusted for repeated observations. All models correct for country-specific differences and time trends.
* p-value < 0·05, ** p-value < 0·01. 1– Adjusted for purchasing power parity and inflation, per capita.

Web Appendix 15: Tax revenues, urbanization and UHC among Low- and Middle-Income Countries, latest available year

**Variables**	Measures of Universal Health Coverage		
	Antenatal coverage[1]	Skilled birth[1]	Formal coverage[2]
	(1)	(2)	(3)
\$100 increase in Tax revenue[3]	5.18** (1.40)	2.34 (1.37)	4.56** (1.39)
1% increase in degree of urbanization[2]	0.49** (0.18)	0.69** (0.17)	0.93** (0.19)
Number of countries	47	43	73
R^2	0.54	0.55	0.55

Notes: World Bank Indicators and WHO. Standard errors are in parentheses and are adjusted for repeated observations. All models correct for country-specific differences and time trends.

* p-value < 0.05, ** p-value < 0.01

1 – Proportion of pregnancies

2 – Proportion of the population

3 – Adjusted for purchasing power parity and inflation, per capita

Web Appendix 16: After correcting for public health spending, private spending and aid, the association of alternative tax regimes with infant mortality, 86 low- and middle-Income countries, 1995-2011

**All countries**	Neonatal mortality[1]	Post-neonatal mortality[2]	Infant (1-5) mortality[3]	Under 5 mortality[4]
	(1)	(2)	(3)	(4)
$100 increase in tax revenue from income, profits, and capital gains (progressive)[5]	-0.095<br>(0.10)	-0.088<br>(0.13)	0.10<br>(0.12)	-0.093<br>(0.29)
$100 increase in tax revenue from goods and services (regressive)[5]	0.081<br>(0.055)	0.15**<br>(0.053)	0.16*<br>(0.065)	0.37*<br>(0.14)
$100 increase in tax revenue from other taxes[5]	0.87<br>(0.52)	0.78<br>(0.58)	0.68<br>(0.49)	2.24<br>(1.47)
$100 increase in public health spending	-0.070<br>(0.36)	0.21<br>(0.47)	-0.022<br>(0.39)	0.10<br>(1.10)
$100 increase in private health spending	-0.58<br>(0.60)	-0.21<br>(0.77)	0.048<br>(0.81)	-0.75<br>(1.90)
$100 increase in ODA	-2.60*<br>(1.06)	-2.97*<br>(1.48)	-3.77*<br>(1.54)	-8.81*<br>(3.49)
Number of countries	86	86	86	86
Country-years	697	697	697	697

Notes: Source: World Bank Indicators and IHME. Standard errors are in parentheses and are adjusted for repeated observations. All models correct for country-specific differences and time trends. 1 – Deaths per 1,000 live births (before age of 1 month); 2 – Deaths per 1,000 per year (before the age of 1); 3 – Deaths per 1,000 per year (after the age of 1 and under the age of 5); 4 – Deaths per 1,000 live births (under the age of 5); 5 – Adjusted for purchasing power parity and inflation, per capita. * p-value < 0·05, ** p-value < 0·01

Web Appendix 17: After correcting for the Great Recession, the association of alternative tax regimes with infant mortality, 86 low- and middle-Income countries, 1995-2011

**All countries**	Neonatal mortality[1]	Post-neonatal mortality[2]	Infant (1-5) mortality[3]	Under 5 mortality[4]
	(1)	(2)	(3)	(4)
$100 increase in tax revenue from income, profits, and capital gains (progressive)[5]	-0.117 (0.103)	-0.0761 (0.127)	0.142 (0.127)	-0.0680 (0.299)
$100 increase in tax revenue from goods and services (regressive)[5]	0.106 (0.0537)	0.179** (0.0567)	0.186** (0.0702)	0.445** (0.152)
$100 increase in tax revenue from other taxes[5]	0.761 (0.505)	0.789 (0.612)	0.759 (0.506)	2.203 (1.483)
Great Recession (1 = 2008-2011)	0.344 (0.191)	0.520* (0.205)	0.551 (0.411)	1.318 (0.678)
Number of countries	86	86	86	86
Country-years	697	697	697	697

Notes: Source: World Bank Indicators and IHME. Standard errors are in parentheses and are adjusted for repeated observations. All models correct for country-specific differences and time trends. 1 – Deaths per 1,000 live births (before age of 1 month); 2 – Deaths per 1,000 per year (before the age of 1); 3 – Deaths per 1,000 per year (after the age of 1 and under the age of 5); 4 – Deaths per 1,000 live births (under the age of 5); 5 – Adjusted for purchasing power parity and inflation, per capita. * p-value < 0·05, ** p-value < 0·01
